# Efficacy of electro-acupuncture and manual acupuncture versus sham acupuncture for knee osteoarthritis: statistical analysis plan for a randomized controlled trial

**DOI:** 10.1186/s13063-019-3513-2

**Published:** 2019-07-04

**Authors:** Jian-Feng Tu, Jing-Wen Yang, Li-Qiong Wang, Yang Wang, Jin-ling Li, Na Zhang, Lu-Lu Lin, Zhang-Sheng Yu, Cun-Zhi Liu

**Affiliations:** 1 School of Acupuncture-Moxibustion and Tuina, Beijing University of Chinese Medicine, 11 Beisanhuan East Road, Chaoyang District, Beijing, 100029 China; 2 Department of Statistics, School of Mathematical Sciences and SJTU-Yale Joint Center for Biostatistics, Shanghai Jiao Tong University, Minhang District, Shanghai, China; 3 Department of Bioinformatics and Biostatistics, School of Life Sciences and Biotechnology and SJTU-Yale Joint Center for Biostatistics, Shanghai Jiao Tong University, Minhang District, Shanghai, China; 4 Department of Statistics, School of Mathematical Sciences, Shanghai Jiao Tong University, Minhang District, Shanghai, China

**Keywords:** Acupuncture, Knee osteoarthritis, Statistical analysis plan

## Abstract

**Background:**

Acupuncture is widely used for knee osteoarthritis (KOA), despite contradictory evidence. This study is designed to determine the efficacy of electro-acupuncture and manual acupuncture versus sham acupuncture for KOA.

**Methods/design:**

This is a multi-center three-arm randomized controlled trial. It will enroll 480 participants with KOA in China. Participants will be randomly assigned 1:1:1 to receive 24 sessions of electro-acupuncture, manual acupuncture, or sham acupuncture over 8 weeks. The primary outcome is the response rate, which is the proportion of patients who achieve the minimal clinically important improvement in pain and function at 8 weeks. The primary outcome will be analyzed using the *Z*-test with the intention-to-treat set. Secondary outcomes include pain, function, global patient assessment, and quality of life. Full details of the statistical analysis plan for the primary and secondary outcomes will be described in this article. The statistical analysis plan was written and submitted without knowledge of the study data.

**Discussion:**

The data will be analyzed according to this pre-specified statistical analysis plan to avoid data-driven analysis and to enhance the transparency of the trial. The aim of the trial is to provide high-quality evidence on the efficacy of acupuncture for KOA.

**Trial registration:**

Clinicaltrials.gov, NCT03366363. Registered on 20 November 2017.

## Background

Knee osteoarthritis (KOA) is one of the leading causes of chronic pain and disability in older adults [1], with symptomatic KOA affecting 8.1% of Chinese people [2] and 1.6–14.9% of Europeans according to age class [3]. The socioeconomic burden of KOA is large, amounting to between 1.0% and 2.5% of the gross domestic product in developed countries [4].

Since no disease-modifying treatment is available, the current management of KOA is symptomatic [5], often with non-steroid anti-inflammatory drugs. However, these have a limited effect [6, 7]. Although total knee replacement is an effective treatment for symptomatic end-stage disease, approximately 15% of patients have continuing pain and mobility problems after surgery and the lifespan of prostheses is limited [8].

Acupuncture is increasingly used in clinical practice [9], although evidence of its efficacy is contradictory [10, 11]. Acupuncture has a dose–effect relationship [12]. However, the dose of acupuncture administered in several previous trials was far from adequate [13]. The frequency of acupuncture is one of the key factors of a dose [14]. A review suggested that the frequency of acupuncture is usually three to five sessions per week in China, whereas it is mostly one session per week in Europe and America [15]. Based on our previous pilot trial [16], high-dose acupuncture (24 sessions in 8 weeks) may be an effective option for KOA. Electro-acupuncture (EA) combines manual acupuncture (MA) with an electric stimulus [17]. Both EA and MA are frequently used in clinical practice. Therefore, the current trial is designed to evaluate the effect of EA and MA, compared with sham acupuncture (SA), in patients with KOA.

The protocol of the trial has been published previously [18] and provides more detail on the trial rationale, eligibility criteria, and interventions. This article aims to report in detail the statistical analysis plan to reduce the risks of reporting bias and enhance the transparency of the trial. The statistical analysis plan was approved on 30 October 2017 (version 1.0) and drafted without knowledge of any of the results.

## Methods/design

### Study design

This three-arm randomized sham-controlled trial has been approved by the ethics committees at each of the nine participating hospitals. Eligible KOA participants, diagnosed according to the American College of Rheumatology criteria [19], are randomly assigned (1:1:1) to receive 24 sessions of EA, MA, or SA over 8 weeks. The block randomization, with block sizes of 6 and 9, is stratified by study center and is performed via a web-based randomization system. Superficial insertion at non-acupoints with no electric current will be used for the SA group, which is one of the most commonly used approaches for administering sham treatments in acupuncture trials. The nature of acupuncture means that acupuncturists are not blinded to treatment allocation; however, patients, outcome assessors, and statisticians will remain masked. Informed consent will be obtained from each participant before randomization. The trial has been registered with ClinicalTrials.gov (NCT03366363).

### Objectives

The objective of the current study is to determine if EA or MA improve clinical outcomes at 8 weeks in patients with KOA. The following two null hypotheses will be tested:

- H1: There is no difference in the patients’ response rate between the EA group and the SA group.
- H2: There is no difference in the patients’ response rate between the MA group and the SA group.

### Outcomes

#### Primary outcome

**Fig. 1:**
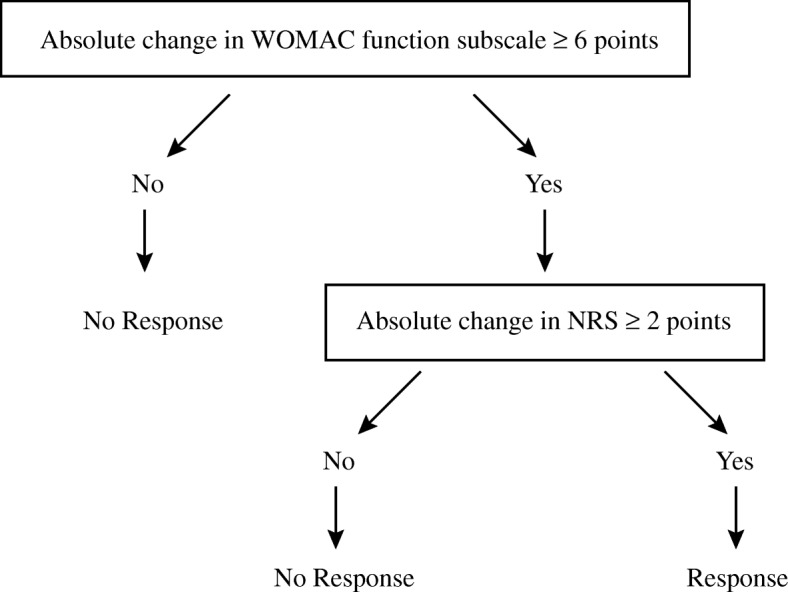
Responder criteria. WOMAC Western Ontario and McMaster Universities Osteoarthritis Index

The primary outcome is the response rate [20], which is the proportion of patients who achieve the minimal clinically important improvement in pain and function at 8 weeks post-randomization. The average pain over the previous week will be assessed using an 11-point numerical rating scale (NRS) [21] with scores ranging from 0 to 10. The minimal clinically important improvement in pain is defined as 2 points in the NRS [11, 22]. The average function over the previous week is measured using the Western Ontario and McMaster Universities Osteoarthritis Index (WOMAC) function subscale [23] with scores ranging from 0 to 68. The minimal clinically important improvement in function is defined as 6 points in the WOMAC function subscale [11, 22]. The criteria for a participant to be a responder are presented in Fig. 1. The response rate is also measured at weeks 4, 16, and 26 after randomization.

#### Secondary outcomes

The secondary outcomes are:

- Numerical rating scale [21]: an 11-point patient-reported outcome measure (PROM) with scores ranging from 0 (no pain) to 10 (worst pain)
- WOMAC [23] pain subscale: a 5-item PROM with total scores ranging from 0 to 20; higher scores indicate worse pain
- WOMAC [23] function subscale: a 17-item PROM with total scores ranging from 0 to 68; lower scores indicate better physical function
- WOMAC [23] stiffness subscale: a 2-item PROM with total scores ranging from 0 to 8; higher scores indicate more stiffness
- Patient global assessment [24]: a 5-point Likert scale
- 12-item Short Form Health Survey (SF-12) [25] physical dimension: total scores range from 0 to 100; lower scores indicate a worse quality of life
- SF-12 [25] mental dimension: total scores range from 0 to 100; higher scores indicate a better quality of life

For the patient global assessment, participants are asked how their knee symptoms were during the past week. Answers can include “extremely improved,” “slightly improved,” “not changed,” “slightly aggravated,” and “extremely aggravated.”

The NRS, WOMAC, patient global assessment, and SF-12 are measured at 4, 8, 16, and 26 weeks after randomization. The blinding assessment is measured at 4 and 8 weeks after randomization. The credibility and expectancy of participants are measured 5 min after the first acupuncture session [26]. The use of rescue medicine is also recorded throughout the trial.

#### Safety outcome

Adverse events are recorded throughout the trial. Based on the potential relationship between acupuncture and adverse events, adverse events are categorized as related to the treatment or not.

### Sample size

Based on the results of our previous trial [16], the response rates for the EA, MA, and SA groups were assumed to be 70%, 60%, and 40%, respectively. With a two-sided significance level of 2.5% and power of 80%, 128 participants in each group will be required to detect a difference as small as 20% between each acupuncture group and the control group. The two-sided significance level of 2.5% is a Bonferroni-adjusted alpha level as per the two predefined primary comparisons: EA vs. SA and MA vs. SA. With an estimated loss-to-follow-up rate of 20%, 480 participants will be recruited in total.

### Statistical analysis

#### Statistical analysis population

A modified full analysis set, a per-protocol set, and a safety set will be used in this trial. The modified full analysis set will consist of all randomized participants who have at least one post-baseline measurement according to the modified intention-to-treat principle. The modified full analysis set will be the primary analysis set, and all analyses will be conducted for this population if not otherwise stated. Analyses of the modified full analysis set will provide an estimate of the effects of EA and MA.

The per-protocol set will include those who complete the treatment and follow-up on time according to the protocol without major violations. Major violations of the protocol will be judged during the blinded audit of the data. They include but are not limited to not meeting the inclusion criteria, meeting the exclusion criteria, receiving other treatments that might affect the symptoms of KOA during the trial, and completing <20 sessions of acupuncture. The per-protocol set will be the secondary analysis set and will be used for the sensitivity analyses.

All those who are randomized and have received at least one session of acupuncture will be defined as the safety set, which is used for the safety analyses.

#### General analysis principles

All data will be summarized by treatment group. Numbers (percentages) will be used to describe categorical data. Either means (standard deviations) or medians (interquartile ranges) will be used for quantitative data depending on whether the variables are normally distributed. If not otherwise stated, the significance level will be set at 0.05. The Bonferroni method will be used to adjust the significance level for multiple comparisons for the primary outcome. The conclusions will be based on the analysis of the primary outcome, and all secondary outcomes will be analyzed to support the primary analysis. All analyses will be carried out using SAS 9.3 (Cary, NC).

#### Descriptive analyses

**Fig. 2:**
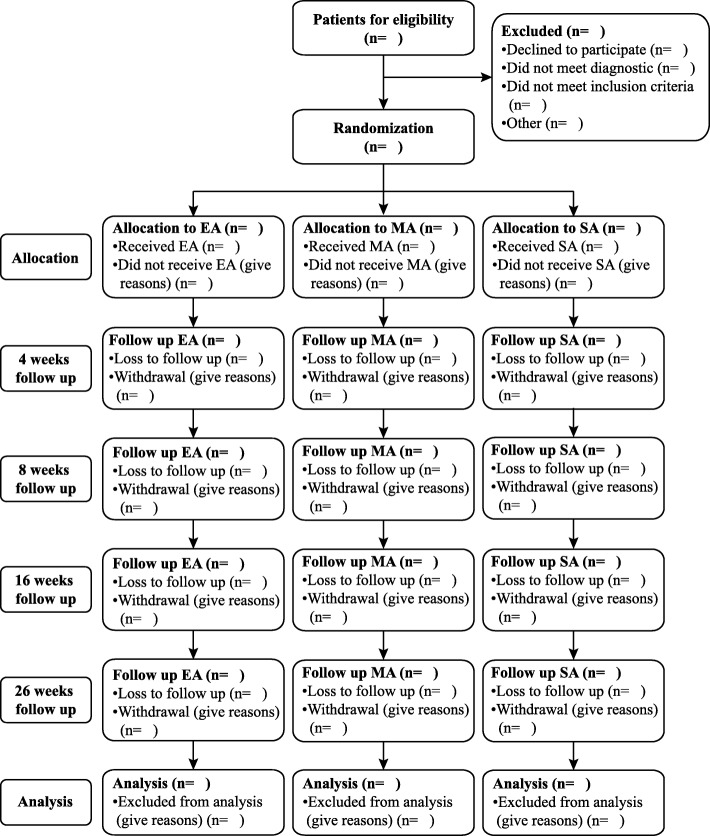
Flow diagram. EA electro-acupuncture, MA manual acupuncture, SA sham acupuncture

The numbers of participants screened, excluded, randomly assigned to each group, interviewed at each follow-up, and analyzed will be summarized using a flow diagram recommended by CONSORT [27] (Fig. 2). Reasons for the losses to follow-up and withdrawals will also be listed by treatment arm.

The demographic characteristics and clinical outcomes at baseline are those presented in Table 1. Missing data for baseline characteristics will not be imputed. Differences among the treatment groups at baseline will not be statistically tested.

**Table 1:** Baseline characteristics

Baseline characteristic	Type	Levels or scale
Gender	Categorical	Male or female
Age	Continuous	Years
Nationality	Categorical	Han or other
Duration of disease	Continuous	Years
Kellgren–Lawrence grade	Categorical	Grade II or III
Body mass index	Continuous	kg/m^2^
Years of education	Categorical	<9, 9–12, or >12
Affected knee	Categorical	Unilateral or bilateral
Previous treatments	Categorical	Injections, medication, physical therapy, acupuncture, exercise, etc.
Concomitant diseases	Categorical	Hypertension, coronary heart disease, diabetes mellitus, hyperlipidemia, etc.
Numerical rating scale	Continuous	Point
WOMAC pain subscale	Continuous	Point
WOMAC function subscale	Continuous	Point
WOMAC stiffness subscale	Continuous	Point
Physical health, SF-12	Continuous	Point
Mental health, SF-12	Continuous	Point

*WOMAC* Western Ontario and McMaster Universities osteoarthritis index, *SF-12* 12-item Short Form Health Survey

#### Analysis of the primary outcome

In the analysis of the primary outcome, the response rates of the three groups at 8 weeks will be calculated and the *Z*-test for comparisons of proportions will be used with the full analysis set. Any missing data at 8 weeks will be imputed using the baseline value. There will be two comparisons. The first comparison is that between the EA group and the SA group. The second is that between the MA group and the SA group. The significance level will be adjusted at 0.025 for multiple comparisons using the Bonferroni method.

#### Analysis of secondary outcomes

For the NRS score, comparisons among the three groups will be assessed by a mixed-effect model with repeated measurement (MMRM) analysis using the NRS score at all follow-up time points as the dependent variable, treatment as the main factor, and the baseline value as a covariate. We set the model as

*y*_*ij*_ = *α* + *u*_*i*_ + *β*_1_time_*ij*_ + *β*_2_treat_*i*_ + *ε*_*ij*_

where *α* is the overall average and *u*_*i*_ is an unknown random effect represented as a subject-specified effect. *β*_1_ and *β*_2_ are unknown fixed effects represented as time and treatment effects, respectively. The covariance matrix *G* is unstructured, and *u*_*i*_~*N*(0, *G*). The random error *ε*_*ij*_~*N*(0, *R*_*i*_). The MMRM for secondary outcomes will be handled by PROC MIXED (SAS). The estimators of unknown parameters will be calculated using an expectation maximalization algorithm, which we expect will converge for the 480 participants in three groups and a single random intercept. If non-convergence does happen, we will consider correcting the initial value, changing the random effect, or using another analysis method like generalized estimating equations. Also, we will test the estimators or models based on a likelihood test and Bayesian information criterion methods.

The modified MMRM also has the center effect and time × treatment effect and it will be used in the sensitivity analysis. The modified MMRM is as follows:

$$ {y}_{ij}=\upalpha +{u}_i+{\upbeta}_1{\mathrm{time}}_{ij}+{\upbeta}_2{\mathrm{treat}}_i+{\upbeta}_3{\mathrm{time}}_{ij}\times {\mathrm{treat}}_i+{\upbeta}_4{\mathrm{center}}_i+{\upvarepsilon}_{ij} $$

where α, u_*i*_, β_1_, β_2_, and ε_*ij*_ are defined as above. β_3_ and β_4_ are unknown fixed effects.

The same approach will be used to analyze the pain, function, and stiffness WOMAC subscales, and SF-12. If normality is violated in the continuous variables, a transformation will be performed before the comparison. A chi-square test will be used for the patient global assessment. These outcomes are shown in Table 2.

**Table 2:** Primary and secondary outcomes

Outcomes	EA (*n*)	MA (*n*)	SAb (*n*)	*P* value	Pairwise comparison					
					EA vs. SA		MA vs. SA		EA vs. MA	
					Difference (95% CI)	*P* value	Difference (95% CI)	*P* value	Difference (95% CI)	*P* value
Success rate, no. (%)										
4 weeks	x (xx.x)	x (xx.x)	x (xx.x)	–	xx.x (xx.x- xx.x)	xx.x	xx.x (xx.x- xx.x)	xx.x	–	–
8 weeks	x (xx.x)	x (xx.x)	x (xx.x)	–	xx.x (xx.x- xx.x)	xx.x	xx.x (xx.x- xx.x)	xx.x	–	–
16 weeks	x (xx.x)	x (xx.x)	x (xx.x)	–	xx.x (xx.x- xx.x)	xx.x	xx.x (xx.x- xx.x)	xx.x	–	–
26 weeks	x (xx.x)	x (xx.x)	x (xx.x)	–	xx.x (xx.x- xx.x)	xx.x	xx.x (xx.x- xx.x)	xx.x	–	–
Numerical Rating Scale, mean (SD)										
Baseline	xx.x (xx.x)	xx.x (xx.x)	xx.x (xx.x)	xx.x	xx.x (xx.x- xx.x)	xx.x	xx.x (xx.x- xx.x)	xx.x	xx.x (xx.x- xx.x)	xx.x
4 weeks	xx.x (xx.x)	xx.x (xx.x)	xx.x (xx.x)		xx.x (xx.x- xx.x)		xx.x (xx.x- xx.x)		xx.x (xx.x- xx.x)	
8 weeks	xx.x (xx.x)	xx.x (xx.x)	xx.x (xx.x)		xx.x (xx.x- xx.x)		xx.x (xx.x- xx.x)		xx.x (xx.x- xx.x)	
16 weeks	xx.x (xx.x)	xx.x (xx.x)	xx.x (xx.x)		xx.x (xx.x- xx.x)		xx.x (xx.x- xx.x)		xx.x (xx.x- xx.x)	
26 weeks	xx.x (xx.x)	xx.x (xx.x)	xx.x (xx.x)		xx.x (xx.x- xx.x)		xx.x (xx.x- xx.x)		xx.x (xx.x- xx.x)	
WOMAC pain subscale, mean (SD)										
Baseline	xx.x (xx.x)	xx.x (xx.x)	xx.x (xx.x)	xx.x	xx.x (xx.x- xx.x)	xx.x	xx.x (xx.x- xx.x)	xx.x	xx.x (xx.x- xx.x)	xx.x
4 weeks	xx.x (xx.x)	xx.x (xx.x)	xx.x (xx.x)		xx.x (xx.x- xx.x)		xx.x (xx.x- xx.x)		xx.x (xx.x- xx.x)	
8 weeks	xx.x (xx.x)	xx.x (xx.x)	xx.x (xx.x)		xx.x (xx.x- xx.x)		xx.x (xx.x- xx.x)		xx.x (xx.x- xx.x)	
16 weeks	xx.x (xx.x)	xx.x (xx.x)	xx.x (xx.x)		xx.x (xx.x- xx.x)		xx.x (xx.x- xx.x)		xx.x (xx.x- xx.x)	
26 weeks	xx.x (xx.x)	xx.x (xx.x)	xx.x (xx.x)		xx.x (xx.x- xx.x)		xx.x (xx.x- xx.x)		xx.x (xx.x- xx.x)	
WOMAC function subscale, mean (SD)										
Baseline	xx.x (xx.x)	xx.x (xx.x)	xx.x (xx.x)	xx.x	xx.x (xx.x- xx.x)	xx.x	xx.x (xx.x- xx.x)	xx.x	xx.x (xx.x- xx.x)	xx.x
4 weeks	xx.x (xx.x)	xx.x (xx.x)	xx.x (xx.x)		xx.x (xx.x- xx.x)		xx.x (xx.x- xx.x)		xx.x (xx.x- xx.x)	
8 weeks	xx.x (xx.x)	xx.x (xx.x)	xx.x (xx.x)		xx.x (xx.x- xx.x)		xx.x (xx.x- xx.x)		xx.x (xx.x- xx.x)	
16 weeks	xx.x (xx.x)	xx.x (xx.x)	xx.x (xx.x)		xx.x (xx.x- xx.x)		xx.x (xx.x- xx.x)		xx.x (xx.x- xx.x)	
26 weeks	xx.x (xx.x)	xx.x (xx.x)	xx.x (xx.x)		xx.x (xx.x- xx.x)		xx.x (xx.x- xx.x)		xx.x (xx.x- xx.x)	
WOMAC stiffness subscale, mean (SD)										
Baseline	xx.x (xx.x)	xx.x (xx.x)	xx.x (xx.x)	xx.x	xx.x (xx.x- xx.x)	xx.x	xx.x (xx.x- xx.x)	xx.x	xx.x (xx.x- xx.x)	xx.x
4 weeks	xx.x (xx.x)	xx.x (xx.x)	xx.x (xx.x)		xx.x (xx.x- xx.x)		xx.x (xx.x- xx.x)		xx.x (xx.x- xx.x)	
8 weeks	xx.x (xx.x)	xx.x (xx.x)	xx.x (xx.x)		xx.x (xx.x- xx.x)		xx.x (xx.x- xx.x)		xx.x (xx.x- xx.x)	
16 weeks	xx.x (xx.x)	xx.x (xx.x)	xx.x (xx.x)		xx.x (xx.x- xx.x)		xx.x (xx.x- xx.x)		xx.x (xx.x- xx.x)	
26 weeks	xx.x (xx.x)	xx.x (xx.x)	xx.x (xx.x)		xx.x (xx.x- xx.x)		xx.x (xx.x- xx.x)		xx.x (xx.x- xx.x)	
Patient global assessment, mean (SD)										
Baseline	xx.x (xx.x)	xx.x (xx.x)	xx.x (xx.x)	xx.x	xx.x (xx.x- xx.x)	xx.x	xx.x (xx.x- xx.x)	xx.x	xx.x (xx.x- xx.x)	xx.x
4 weeks	xx.x (xx.x)	xx.x (xx.x)	xx.x (xx.x)		xx.x (xx.x- xx.x)		xx.x (xx.x- xx.x)		xx.x (xx.x- xx.x)	
8 weeks	xx.x (xx.x)	xx.x (xx.x)	xx.x (xx.x)		xx.x (xx.x- xx.x)		xx.x (xx.x- xx.x)		xx.x (xx.x- xx.x)	
16 weeks	xx.x (xx.x)	xx.x (xx.x)	xx.x (xx.x)		xx.x (xx.x- xx.x)		xx.x (xx.x- xx.x)		xx.x (xx.x- xx.x)	
26 weeks	xx.x (xx.x)	xx.x (xx.x)	xx.x (xx.x)		xx.x (xx.x- xx.x)		xx.x (xx.x- xx.x)		xx.x (xx.x- xx.x)	
SF-12 physical health, mean (SD)										
Baseline	xx.x (xx.x)	xx.x (xx.x)	xx.x (xx.x)	xx.x	xx.x (xx.x- xx.x)	xx.x	xx.x (xx.x- xx.x)	xx.x	xx.x (xx.x- xx.x)	xx.x
4 weeks	xx.x (xx.x)	xx.x (xx.x)	xx.x (xx.x)		xx.x (xx.x- xx.x)		xx.x (xx.x- xx.x)		xx.x (xx.x- xx.x)	
8 weeks	xx.x (xx.x)	xx.x (xx.x)	xx.x (xx.x)		xx.x (xx.x- xx.x)		xx.x (xx.x- xx.x)		xx.x (xx.x- xx.x)	
16 weeks	xx.x (xx.x)	xx.x (xx.x)	xx.x (xx.x)		xx.x (xx.x- xx.x)		xx.x (xx.x- xx.x)		xx.x (xx.x- xx.x)	
26 weeks	xx.x (xx.x)	xx.x (xx.x)	xx.x (xx.x)		xx.x (xx.x- xx.x)		xx.x (xx.x- xx.x)		xx.x (xx.x- xx.x)	
SF-12 mental health, mean (SD)										
Baseline	xx.x (xx.x)	xx.x (xx.x)	xx.x (xx.x)	xx.x	xx.x (xx.x- xx.x)	xx.x	xx.x (xx.x- xx.x)	xx.x	xx.x (xx.x- xx.x)	xx.x
4 weeks	xx.x (xx.x)	xx.x (xx.x)	xx.x (xx.x)		xx.x (xx.x- xx.x)		xx.x (xx.x- xx.x)		xx.x (xx.x- xx.x)	
8 weeks	xx.x (xx.x)	xx.x (xx.x)	xx.x (xx.x)		xx.x (xx.x- xx.x)		xx.x (xx.x- xx.x)		xx.x (xx.x- xx.x)	
16 weeks	xx.x (xx.x)	xx.x (xx.x)	xx.x (xx.x)		xx.x (xx.x- xx.x)		xx.x (xx.x- xx.x)		xx.x (xx.x- xx.x)	
26 weeks	xx.x (xx.x)	xx.x (xx.x)	xx.x (xx.x)		xx.x (xx.x- xx.x)		xx.x (xx.x- xx.x)		xx.x (xx.x- xx.x)	

*EA* electro-acupuncture, *MA* manual acupuncture, *SA* sham acupuncture, *WOMAC* Western Ontario and McMaster Universities Osteoarthritis Index, *SD* standard deviation, *SF-12* 12-item Short Form Health Survey

#### Safety analyses

Based on the potential relationship between acupuncture and adverse events, adverse events are categorized as treatment-related or not. Acupuncture-related adverse events will be summarized by group and compared using a chi-square test (or Fisher’s exact test).

#### Blinding analyses

A kappa analysis will be used to determine whether participants correctly guessed their group assignment at a higher rate than would be expected by chance.

#### Additional analyses

Another three schemes to deal with any missing data for the primary outcome will be carried out to examine the robustness of the conclusion. First, any data missing at 8 weeks will be imputed using the last observation carried forward approach. Second, we will directly remove any missing data. Third, any data missing at 8 weeks will be imputed using multiple imputation [28]. By assuming the data missing are random, the missing data will be imputed using the Monte Carlo Markov chain method for multiple imputation with Proc MI (SAS). The initial seed will be set at 1000 and five datasets will be imputed. Any data missing for the primary outcome will be imputed by the observation value of age, gender, body mass index, Kellgren–Lawrence grade, and duration of disease. A subgroup analysis based on Kellgren–Lawrence grade will be performed.

## Discussion and trial status

The trial will provide high-quality evidence of the efficacy of EA and MA for KOA. This paper provides details of the planned statistical analyses for the current trial and will help to reduce the risks of outcome reporting bias and data-driven results [29]. This paper has been prepared in accordance with the published guidelines for the content of statistical analysis plans [30]. As of October 2018, 480 patients from nine centers had been randomized. The final date of follow-up is April 7, 2019. This analysis plan was written prior to completion of the trial data collection phase.

## Data Availability

No original data are currently available relating to the detailed statistical analysis plan. The datasets used during the current study will be available from the corresponding author on reasonable request.

## Abbreviations

ANOVA: One-way analysis of variance
EA: Electro-acupuncture
KOA: Knee osteoarthritis
MA: Manual acupuncture
MMRM: Mixed-effect model with repeated measurement
NRS: Numerical rating scale
PROM: Patient-reported outcome measure
SA: Sham acupuncture
SF-12: 12-item Short Form Health Survey
WOMAC: Western Ontario and McMaster Universities Osteoarthritis Index
